# Serum Maresin-1 in Type 2 Diabetes: A Biomarker Profile in Relation to Diabetic Retinopathy Phenotypes and Proteinuria

**DOI:** 10.3390/diagnostics16142287

**Published:** 2026-07-22

**Authors:** Mustafa Timurkaan, Esra Suay Timurkaan, Muhammed Fuad Uslu, Fatih Cem Gül, Hakan Ayyıldız

**Affiliations:** 1 Department of Internal Medicine, Elazig Fethi Sekin City Hospital, 23280 Elazig, Turkey; dresrasuay@gmail.com (E.S.T.); dr.fuslu@gmail.com (M.F.U.); 2 Department of Ophthalmology, Elazig Fethi Sekin City Hospital, 23280 Elazig, Turkey; fatihcemgul@gmail.com; 3 Department of Biochemistry, Elazig Fethi Sekin City Hospital, 23280 Elazig, Turkey; hknayyildiz@hotmail.com

**Keywords:** maresin-1, type 2 diabetes mellitus, diabetic retinopathy, proliferative diabetic retinopathy, non-proliferative diabetic retinopathy, proteinuria

## Abstract

**Background/Objectives:** The clinical profile of serum Maresin-1 (MaR1) in relation to diabetic retinopathy phenotypes and proteinuria in type 2 diabetes mellitus (T2DM) remains unclear. We evaluated serum MaR1 across healthy controls and patients with T2DM without diabetic retinopathy (DR), non-proliferative DR (NPDR), or proliferative DR (PDR), and examined the relationship between MaR1 and the urine protein-to-creatinine ratio (UPCR). **Methods:** This single-center cross-sectional study included 93 participants. Serum MaR1 was measured by ELISA. Group differences were assessed with the Kruskal–Wallis test and Holm-adjusted post hoc tests. DR phenotypes were analyzed among patients with T2DM. The MaR1-UPCR relationship was examined using correlation and adjusted regression models. **Results:** MaR1 differed across groups (H = 49.36, *p* < 0.001, epsilon^2^ = 0.521). The median MaR1 was 89.8 (82.8–97.2) pg/mL in controls and 34.3 (33.0–36.0), 35.8 (34.4–36.7), and 34.0 (33.1–35.7) pg/mL in T2DM without DR, NPDR, and PDR, respectively. MaR1 was higher in controls than in all T2DM groups, whereas T2DM groups did not differ. Within T2DM, MaR1 was not associated with DR stage (H = 4.44, *p* = 0.109; rho = −0.001, *p* = 0.996). MaR1 was inversely related to the UPCR overall (rho = −0.272, *p* = 0.008), but not within T2DM (rho = −0.057, *p* = 0.634) or in adjusted models. **Conclusions:** MaR1 was markedly lower in T2DM than in controls. This reduction was not explained by DR stage or proteinuria. These findings indicate that MaR1 should be interpreted as a T2DM-associated systemic alteration rather than as a marker of retinopathy stage or proteinuria.

## 1. Introduction

Diabetic retinopathy (DR) and diabetic kidney disease (DKD) are major microvascular complications of type 2 diabetes mellitus (T2DM). They are central targets of contemporary diabetes care because of their links with vision loss, renal function decline, and long-term morbidity [1,2]. Hyperglycemia, hypertension, and metabolic dysregulation remain key clinical determinants of these complications. However, diabetic microvascular injury is not only a passive consequence of chronic metabolic exposure. Oxidative stress, vascular dysfunction, and chronic inflammation are important components of this biological process [3,4].

Maresin-1 (MaR1) is a docosahexaenoic acid-derived lipid mediator in the specialized pro-resolving mediator family, a class of endogenous molecules that help terminate inflammation and restore tissue homeostasis. It has been linked to inflammatory resolution, macrophage-mediated resolution, tissue repair, and regenerative responses [3,4,5,6]. Experimental and translational studies suggest that MaR1 may be related to adipose tissue inflammation, insulin signaling, glucose uptake, and impaired reparative macrophage responses in the diabetic milieu [7,8,9]. However, these data do not show that serum MaR1 levels directly reflect clinical complication stages in humans.

Human data on MaR1 in diabetes are limited but clinically relevant. In patients with T2DM and diabetic foot ulcer, lower plasma MaR1 levels have been reported. Lower MaR1 levels have also been linked to obesity, metabolic impairment, and insulin resistance [10]. In another study of individuals without diabetes or chronic kidney disease, serum MaR1 levels were lower in overweight and obese groups and were inversely related to insulin resistance indices [11]. From the renal perspective, the literature is more heterogeneous. Studies of serum and urinary MaR1 have reported different patterns in relation to DKD, diabetic nephropathy, albuminuria, and renal function markers [12,13,14]. These findings suggest that the behavior of MaR1 in diabetic complications may depend on the biological matrix, disease stage, and patient selection.

In the retinal field, bioactive lipid mediators have been linked to pathological angiogenesis and inflammatory retinal injury. However, human data specific to MaR1 remain limited [15]. A recent study reported lower plasma MaR1 levels and higher aqueous humor MaR1 levels in patients with proliferative diabetic retinopathy (PDR). That study did not include the non-proliferative diabetic retinopathy (NPDR) phenotype [16]. Therefore, the behavior of serum MaR1 across the clinical spectrum from healthy controls to T2DM without DR, NPDR, and PDR remains unclear. Retinal and renal microvascular complications are also evaluated together in clinical diabetes care. For this reason, examining whether proteinuria is related to serum MaR1 is rational in this setting [17,18].

This study was designed to extend the limited data on the clinical profile of serum MaR1 in human T2DM-related microvascular complications. We evaluated serum MaR1 levels in healthy controls, patients with T2DM without DR, patients with NPDR, and patients with PDR. We also examined whether serum MaR1 was related to the urine protein-to-creatinine ratio (UPCR) within this retinal microvascular framework.

## 2. Materials and Methods

### 2.1. Study Design and Participants

This was a single-center, observational, cross-sectional study in which participants were prospectively enrolled. The study was reported in accordance with the Strengthening the Reporting of Observational Studies in Epidemiology (STROBE) statement [19]. It included adults with type 2 diabetes mellitus (T2DM) and healthy controls. The study was jointly conducted by the Departments of Internal Medicine and Ophthalmology and the Biochemistry Laboratory at Elazig Fethi Sekin City Hospital. The study population consisted of adult patients with a previous diagnosis of T2DM who attended the Internal Medicine outpatient clinic between 1 June 2025 and 31 August 2025 for routine follow-up. Healthy individuals evaluated in the same clinical setting for control purposes were also included.

The inclusion criteria were age older than 18 years, voluntary participation in the outpatient evaluation, and written informed consent. For the T2DM groups, eligibility also required a previous clinical diagnosis of T2DM. The exclusion criteria were type 1 diabetes, gestational diabetes, insulin therapy, known chronic inflammatory disease, active or previous malignancy, advanced heart failure, advanced liver disease, advanced-stage kidney disease, thyroid dysfunction, immunosuppressive treatment, chronic steroid use, recent surgery, pregnancy, and any disease requiring active psychiatric treatment. Patients were also excluded from the retinopathy phenotype analysis if the ophthalmologic assessment showed an additional ocular pathology that prevented reliable DR classification, inadequate fundus evaluation, or retinal findings not attributable to diabetes.

All patients with T2DM were evaluated by an ophthalmologist to determine the presence and stage of diabetic retinopathy. Retinopathy phenotype was classified as no DR, non-proliferative DR (NPDR), or proliferative DR (PDR), based on clinical ophthalmologic assessment and available ophthalmologic records. The ophthalmologic assessment also aimed to exclude non-diabetic ocular conditions that could affect the reliability of retinopathy classification. Patients were not assigned to study groups if fundus evaluation was inadequate, if retinal findings could be explained by a non-diabetic ocular disease, or if the DR phenotype could not be classified reliably. The healthy control group consisted of individuals who attended the Internal Medicine outpatient clinic for control purposes and remained eligible after the exclusion criteria were applied.

To reduce treatment-related heterogeneity, only patients with T2DM receiving non-insulin oral antidiabetic treatment were considered eligible. Eligible regimens included metformin, sulfonylureas, and dipeptidyl peptidase-4 inhibitors. Patients receiving insulin therapy, glucagon-like peptide-1 receptor agonists, or sodium-glucose cotransporter-2 inhibitors were excluded. In addition, participants with known hypertension or use of antihypertensive treatment, including angiotensin-converting enzyme inhibitors or angiotensin receptor blockers, were not included. This restriction reduced heterogeneity related to medications that could affect proteinuria.

Clinical data and biological samples were initially collected from all volunteer participants. The analysis set was then narrowed after the exclusion criteria were applied and the risk of misclassification was reduced. Thirteen participants voluntarily withdrew from the study. Two participants were later found to be pregnant. Three participants had active or previous malignancy. These participants were excluded from the analysis. Three individuals planned for inclusion in the healthy control group were also removed from the study population because additional comorbidities were identified under the exclusion criteria. After all exclusion criteria were applied, the final analysis dataset included 93 participants.

Participants in the final analysis set were assigned to four predefined groups: healthy controls, T2DM without DR, T2DM with NPDR, and T2DM with PDR. The primary analytic framework was the comparison of serum MaR1 levels across these four groups. Proteinuria was evaluated as a renal correlate of serum MaR1, not as a primary outcome expected to increase across DR stages. Healthy controls were included as a clinically meaningful reference group. They were not included in retinopathy phenotype analyses performed among patients with T2DM.

### 2.2. Ethical Approval and Informed Consent

The study protocol was approved by the Ethics Committee of Elazig Fethi Sekin City Hospital (date: 23 May 2024; decision no: 2024/08-30). The study was conducted in accordance with the principles of the Declaration of Helsinki. Written informed consent was obtained from all participants before enrollment.

### 2.3. Clinical Group Definitions and Retinopathy Assessment

All participants with T2DM underwent a comprehensive ophthalmic examination by a retinal specialist. Diabetic retinopathy was graded according to the International Clinical Diabetic Retinopathy Disease Severity Scale [20]. Grading was based primarily on fundus examination, while optical coherence tomography and fundus fluorescein angiography were performed when clinically indicated to confirm the diagnosis and assess disease severity. Based on these assessments, patients were categorized as T2DM without DR, T2DM with NPDR, or T2DM with PDR. The primary four-level study group variable comprised healthy controls, T2DM without DR, T2DM with NPDR, and T2DM with PDR.

In supportive analyses performed among participants with T2DM, retinopathy phenotype was evaluated as a three-level ordinal variable: no DR, NPDR, and PDR. Healthy controls were not included in these analyses. This approach was used to avoid misinterpreting the healthy control–T2DM difference as an effect of DR stage.

### 2.4. Clinical and Biochemical Measurements

Age, sex, medical history, body weight, and height were recorded for all participants. Height and body weight were measured using calibrated devices. Body mass index (BMI) was calculated as body weight in kilograms divided by height in meters squared. Blood pressure was measured in the morning according to a standard measurement protocol.

Venous blood samples were collected into BD Vacutainer SST™ II Advance tubes for serum separation. After centrifugation, aprotinin was added to serum aliquots to inhibit proteolysis. Serum samples were aliquoted into low-binding tubes, kept on ice during processing, and stored at −20 °C until analysis. Samples were processed to allow for only one freeze–thaw cycle.

Fasting glucose, urea, creatinine, total cholesterol, HDL cholesterol, LDL cholesterol, and triglyceride levels were measured using an AU 5800 analyzer (Beckman Coulter, Inc., Miami, FL, USA). HbA1c was measured by capillary zone electrophoresis (Sebia Capillary 3 Tera, Lisses, France). Biochemical variables also included fasting blood glucose, C-peptide, urea, creatinine, spot urine protein, and urine creatinine. The urine protein-to-creatinine ratio (UPCR) was determined from a single first-morning urine sample obtained from each participant. Urinary protein and urinary creatinine concentrations were measured in mg/dL. Because both concentrations were measured in the same units, the UPCR was calculated as their quotient and reported as a unitless ratio in this study.

### 2.5. Serum Maresin-1 Measurement

Serum MaR1 concentrations were expressed as pg/mL. Serum MaR1 levels were measured using a commercially available ELISA kit according to the manufacturer’s protocol (Bioassay Technology Laboratory [BT Lab], Catalog No. E7050Hu, Shanghai, China). The assay standard curve ranged from 37.5 to 2400 pg/mL, with a sensitivity of 21.23 pg/mL. The manufacturer-reported intra-assay and inter-assay coefficients of variation were <10% and <12%, respectively. Samples were analyzed as single measurements in a single analytical run by operators blinded to the clinical data. A 4800 pg/mL stock calibrator was serially diluted to generate the standard curve, and a zero standard was included in the analytical run. Absorbance values were read using a Chromate 4300 microplate reader (Awareness Technology, Palm City, FL, USA). Serum MaR1 measurements were used for the primary comparison across the four study groups and for supportive analyses related to proteinuria.

### 2.6. Analytical Framework

The primary outcome was serum MaR1 level across the four predefined study groups. Secondary and supportive analyses evaluated the relationship between serum MaR1 and UPCR, retinopathy phenotype comparisons among participants with T2DM, and correlations between MaR1 and selected renal, metabolic, and clinical variables. Renal assessment in this study was limited to UPCR-based proteinuria.

### 2.7. Statistical Analysis

All statistical analyses were performed using R version 4.5.1 in the RStudio environment. Continuous variables were summarized as mean (SD) or median (IQR), according to their distribution. Categorical variables were presented as *n* (%). Baseline clinical and biochemical characteristics were compared across the four study groups using Welch’s ANOVA for age, one-way ANOVA for LDL cholesterol and total cholesterol, the Kruskal–Wallis test for non-normally distributed continuous variables, and Pearson’s chi-square test for sex distribution. Diabetes duration was compared only across the three T2DM groups.

The primary comparison of serum MaR1 across the four study groups was performed using the Kruskal–Wallis test. Effect size was reported as epsilon-squared. Pairwise post hoc comparisons were performed using Dunn-type rank-based tests with Holm adjustment. In the supportive analysis among participants with T2DM, serum MaR1 levels were compared across no DR, NPDR, and PDR phenotypes using the Kruskal–Wallis test and Dunn–Holm post hoc comparisons. The ordinal relationship between DR stage and serum MaR1 was assessed using Spearman’s rank correlation.

The relationship between serum MaR1 and UPCR was assessed using Spearman rank correlation in the whole cohort and in the T2DM-only analysis population. Additional correlations between serum MaR1 and selected renal, metabolic, and clinical variables were also examined using Spearman rank correlation. Multiple comparisons were controlled using the Benjamini–Hochberg false discovery rate method.

Supportive linear regression models were fitted to evaluate the association between the UPCR and serum MaR1. Serum MaR1 was used as the dependent variable, and the UPCR was entered into the model per 1-SD increase. In the whole cohort, models were fitted as unadjusted, adjusted for age, sex, and BMI, and adjusted for age, sex, BMI, and study group. In the T2DM-only analysis population, models were fitted as unadjusted, adjusted for age, sex, BMI, HbA1c, and diabetes duration, and additionally adjusted for DR phenotype. Model diagnostics included the Shapiro–Wilk test for residual normality, the Breusch–Pagan test for heteroscedasticity, and Cook’s distance for influential observations. Because heteroscedasticity and influential observations were identified, regression inference was based on HC3 robust standard errors. These regression models were considered supportive and were not used to replace the primary non-parametric analyses. Regression models using log-transformed MaR1 were also examined as sensitivity analyses. Log transformation did not adequately improve the residual diagnostics and did not materially alter the inference regarding the association between UPCR and MaR1. Raw MaR1 was therefore retained to preserve interpretability on the original pg/mL scale.

A supportive group-adjusted model was also fitted with serum MaR1 as the dependent variable and study group, age, sex, and BMI as independent variables. This model was used to examine whether the healthy control–T2DM group contrast persisted after accounting for basic baseline covariates. The study was not designed for diagnostic or predictive model development. Therefore, receiver operating characteristic analysis, cutoff estimation, decision curve analysis, net reclassification improvement, integrated discrimination improvement, machine learning, stepwise regression, and arbitrary dichotomization of proteinuria were not performed. Log transformation was not automatically used as the main analysis. For all tests, a *p*-value < 0.05 was considered statistically significant.

## 3. Results

### 3.1. Study Population and Baseline Characteristics

The analysis dataset included 93 participants: 20 healthy controls, 32 patients with T2DM without DR, 21 patients with T2DM with NPDR, and 20 patients with T2DM with PDR. All participants met the BMI > 18 kg/m^2^ criterion. The T2DM-only analysis set included 73 participants. Baseline clinical and biochemical characteristics are shown in Table 1. Age, diabetes duration, fasting glucose, HbA1c, triglycerides, urea, and UPCR differed across groups. Sex distribution and BMI did not differ significantly across the four groups.

### 3.2. Primary Analysis: Serum Maresin-1 Across Healthy Controls and Diabetic Retinopathy Phenotypes

**Figure 1:**
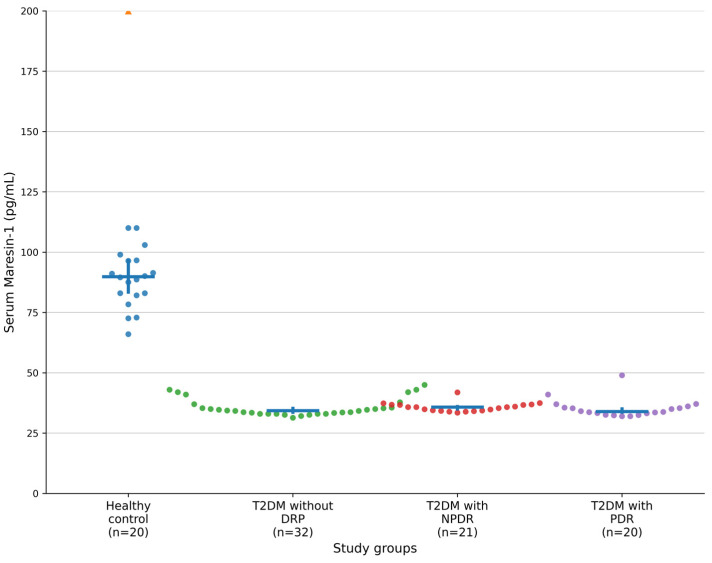
Serum Maresin-1 levels across the four study groups. Jittered individual data points are shown. Horizontal bars represent the median, and vertical bars represent the interquartile range. The y-axis was limited for visual clarity; full ranges are provided in Table 2.

Serum MaR1 levels differed significantly across the four predefined groups (Krus-kal–Wallis H = 49.36, *p* < 0.001, epsilon^2^ = 0.521). The median serum MaR1 was 89.8 (82.8–97.2) pg/mL in healthy controls, 34.3 (33.0–36.0) pg/mL in the T2DM without DR group, 35.8 (34.4–36.7) pg/mL in the T2DM with NPDR group, and 34.0 (33.1–35.7) pg/mL in the T2DM with PDR group (Table 2; Figure 1). Serum Maresin-1 differed across the four predefined groups (Kruskal–Wallis H = 49.36, *p* < 0.001, epsilon-squared = 0.521). Holm-adjusted post hoc comparisons showed higher serum MaR1 levels in healthy controls than in each T2DM subgroup. Pairwise differences among the three T2DM phenotypes were not statistically significant (Table 3).

### 3.3. Supportive T2DM-Only Retinopathy Phenotype Analysis

Among participants with T2DM, serum MaR1 levels did not differ significantly across no DR, NPDR, and PDR phenotypes (Kruskal–Wallis H = 4.44, *p* = 0.109, epsilon^2^ = 0.035). The ordinal relationship between DR stage and serum MaR1 was not significant (Spearman rho = −0.001, *p* = 0.996). Detailed group summaries and pairwise comparisons are shown in Appendix Table A1 and Table A2.

### 3.4. Maresin-1 and Proteinuria

**Figure 2:**
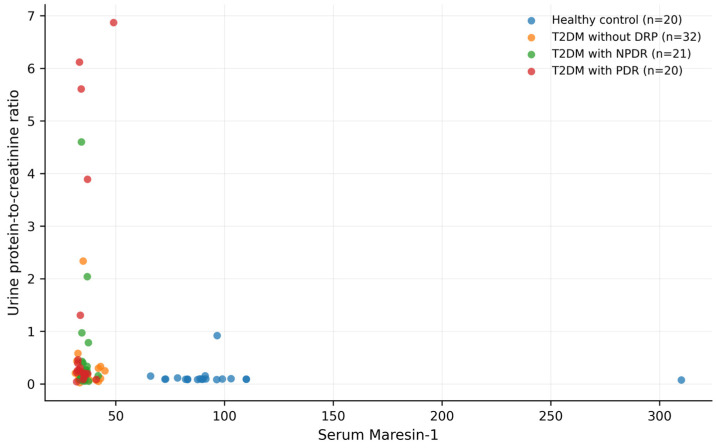
Association between serum Maresin-1 and urine protein-to-creatinine ratio.

In the whole cohort, serum MaR1 was inversely correlated with UPCR (Spearman rho = −0.272, *p* = 0.008). This association was not observed in the T2DM-only analysis set (Spearman rho = −0.057, *p* = 0.634) (Table 4, Figure 2). In exploratory correlations within this population, serum MaR1 showed no FDR-adjusted significant association with selected renal, metabolic, or clinical variables (Table 5).

### 3.5. Supportive Adjusted Models

In supportive regression models, the UPCR was not independently associated with serum MaR1. This finding was consistent in the whole cohort and in the T2DM-only population after adjustment for age, sex, BMI, study group, HbA1c, diabetes duration, and DR phenotype where appropriate (Table 6).

In the supportive group model adjusted for age, sex, and BMI, serum MaR1 levels were lower in each T2DM subgroup than in healthy controls. The adjusted difference was −62.64 pg/mL for T2DM without DR, −61.07 pg/mL for T2DM with NPDR, and −61.73 pg/mL for T2DM with PDR; all were significant at *p* < 0.001. In this model, age, sex, and BMI were not significantly associated with MaR1 (Table 7).

## 4. Discussion

The main finding of this cross-sectional study was that serum MaR1 levels were markedly higher in healthy controls than in the T2DM subgroups. However, this difference did not appear as a stepwise increase or decrease across retinopathy severity. When participants with T2DM were evaluated separately, serum MaR1 levels did not differ among the groups without DR, with NPDR, and with PDR. No ordinal association with DR stage was observed. Similarly, the inverse association between MaR1 and the urine protein-to-creatinine ratio (UPCR) in the whole cohort was not retained in the T2DM-only analysis or in supportive adjusted models. These findings suggest that serum MaR1 reflects a systemic difference associated with T2DM rather than an independent marker of DR stage or proteinuria in this cohort.

The finding of lower MaR1 levels in T2DM phenotypes compared to healthy controls is broadly consistent with reports of reduced MaR1 in metabolic and diabetic phenotypes. Miao et al. reported lower plasma MaR1 levels in T2DM, particularly in patients with diabetic foot ulcer [10]. Deniz et al. showed that serum MaR1 levels were lower in overweight and obese individuals and were inversely related to insulin resistance indices [11]. Li et al. also reported lower serum MaR1 levels in the context of T2DM and diabetic kidney disease (DKD) [12]. The present study adds to this clinical context by showing a similar healthy control–T2DM separation in a cohort structured around DR phenotypes. However, this pattern should not be interpreted as a DR-specific finding. The analyses restricted to patients with T2DM did not show a significant separation across retinopathy stages.

This distinction defines the main interpretation of the study. A biomarker may separate healthy and diabetic states. This does not mean that the same biomarker can distinguish diabetic complication phenotypes. Our data support this more specific interpretation. Serum MaR1 showed a clear contrast between healthy controls and T2DM. It did not show a gradient from no DR to NPDR and PDR. Therefore, the present findings do not support serum MaR1 as a marker of DR severity within a defined T2DM cohort. A more accurate interpretation is that the systemic diabetic milieu may be associated with lower MaR1 levels, whereas retinopathy stage did not generate a stable or additional serum MaR1 signal in this sample.

This finding is partly consistent with the closest human DR-MaR1 study, but it requires a broader interpretive boundary. Gungor Kobat et al. reported lower plasma MaR1 levels and higher aqueous humor MaR1 levels in PDR [16]. In the present study, serum MaR1 levels were also lower in the T2DM subgroups, including PDR, than in healthy controls. However, among participants with T2DM, serum MaR1 levels did not differ significantly among the groups without DR, with NPDR, and with PDR. These findings suggest that systemic MaR1 reduction may be related to T2DM, but that serum MaR1 does not behave as a marker that increases or decreases across DR stage. The previous study did not include an NPDR group and evaluated plasma and aqueous humor rather than serum. Therefore, serum, plasma, and intraocular MaR1 levels should not be considered directly equivalent. Taken together, these differences extend the previous findings and show that systemic MaR1 levels cannot be assumed to directly reflect local intraocular pro-resolving lipid mediator activity.

Preclinical retinal data provide biological plausibility. They do not remove the limits of clinical interpretation. In ARPE-19 models, MaR1 has been reported to reduce high glucose-induced ferroptosis by activating the Nrf2/HO-1/GPX4 pathway [21]. In human retinal pigment epithelial cells, MaR1 has also been reported to attenuate high glucose-induced pyroptosis through autophagy linked to SIRT1/PPAR-gamma signaling [22]. These findings support the biological relevance of MaR1 in retinal cellular injury models. However, they do not show that a single serum MaR1 measurement reflects retinal tissue activity or clinical DR stage in humans. The broader literature on bioactive lipids in pathological retinopathy also supports a role for lipid mediators in retinal vascular and inflammatory responses. It does not validate circulating MaR1 as a staging biomarker for DR [15].

The proteinuria analyses defined an important boundary. They showed that the serum MaR1 finding cannot be directly reduced to renal protein loss. Although serum MaR1 was inversely correlated with UPCR in the whole cohort, this association disappeared when the analysis was restricted to participants with T2DM. Supportive adjusted regression models were therefore used to assess the robustness of this null finding after accounting for potential confounders, including DR phenotype, and likewise showed no independent association between MaR1 and UPCR. Together, these findings suggest that the whole-cohort correlation may have been influenced by the broader clinical separation associated with T2DM status. This finding suggests that the whole-cohort correlation may have been influenced by the broader clinical separation associated with T2DM status. More importantly, within the T2DM group, serum MaR1 levels did not change in a parallel, independent, and consistent manner with proteinuria. The present data therefore support serum MaR1 as a more general systemic difference associated with T2DM rather than as a renal biomarker reflecting proteinuria in this cohort.

Based on our data, this interpretation is consistent with the heterogeneity of the renal MaR1 literature. Li et al. reported lower serum MaR1 levels in DKD and an inverse association with the urinary albumin-to-creatinine ratio. In contrast, Bulu et al. reported higher serum MaR1 levels in diabetic nephropathy and opposite clinical correlations with markers of renal dysfunction [12,13]. Morita et al. examined urinary lipid mediators and reported lower urinary MaR1 levels in more advanced diabetic nephropathy stages. However, urinary MaR1 cannot be considered directly equivalent to serum or plasma MaR1 [14]. These differences suggest that MaR1 findings in diabetic renal disease may depend on biological matrix, renal phenotype, disease stage, and patient selection. In the present study, proteinuria was evaluated as a renal correlate in a DR-focused cohort, not as a primary phenotype enriched for DKD.

Evaluating proteinuria in a DR-focused MaR1 study is clinically meaningful because retinal and renal involvement overlap in diabetic microvascular disease. Previous studies have shown that albuminuria is associated with more severe DR and that retinopathy is linked to worse renal prognosis in proteinuric T2DM populations [17,18]. These data provide a clinical rationale for considering renal variables in biomarker studies structured around DR phenotypes. However, this clinical relationship does not mean that MaR1 reflects retinal and renal injury through a shared biological axis. In the present cohort, serum MaR1 was not independently and consistently related to proteinuria within T2DM. These data do not support positioning MaR1 as a shared biomarker of retinal-renal injury.

Experimental renal studies support the biological relevance of MaR1 in diabetic kidney injury. MaR1 has been shown to reduce inflammatory and fibrotic responses in high glucose-induced glomerular mesangial cell injury [23]. Li et al. also reported that MaR1 may attenuate DKD through the LGR6-mediated cAMP-SOD2 antioxidant pathway in experimental systems [12]. These data point to a potential protective role for MaR1 in renal cellular stress, inflammation, and oxidative injury. However, in the present clinical cohort, serum MaR1 levels did not show a parallel and independent change with proteinuria within T2DM. Our study therefore does not refute experimental renal MaR1 biology. Rather, it shows that serum MaR1 should not be interpreted as a direct clinical indicator of proteinuria in T2DM.

This study has several strengths. Serum MaR1 levels were evaluated within a predefined clinical group structure extending from healthy controls to T2DM without DR, NPDR, and PDR. This design allowed us to examine whether the main MaR1 difference occurred only between healthy controls and T2DM, or whether MaR1 also changed with retinopathy stage within T2DM. The inclusion of an NPDR group allowed retinopathy to be evaluated across a more balanced clinical spectrum, rather than only by absence and advanced disease. In addition, the four-group comparison was paired with a T2DM-only DR phenotype analysis after healthy controls were excluded. This approach helped distinguish whether lower serum MaR1 was related to DR stage or to the broader presence of T2DM. Including proteinuria in the same analytic framework also allowed us to assess whether MaR1 showed a clinical pattern related to renal microvascular variables, beyond retinal phenotypes alone.

The limitations of the study should also be considered. The cross-sectional design does not allow for causal or temporal interpretation. Serum MaR1 was measured at a single time point. No a priori sample size calculation or formal power analysis was performed because recruitment was conducted within the fixed period specified in the study approval, and the sample size was determined by the number of eligible consenting participants enrolled during this period. Consequently, the study may have had limited power to detect smaller but clinically meaningful differences in serum MaR1 among the T2DM retinopathy phenotypes, and the null findings should be interpreted cautiously. Serum levels cannot be assumed to directly reflect intraocular, urinary, or renal tissue MaR1 activity. Inflammatory biomarkers, including C-reactive protein and cytokines, were not measured. Consequently, this study could not determine whether the lower serum MaR1 levels observed in the T2DM groups were associated with systemic inflammatory activity. The healthy control group provided a clinically meaningful reference stratum, but its age distribution did not fully overlap with the T2DM groups. Therefore, residual confounding related to age and metabolic status cannot be completely excluded. Treatment heterogeneity, the duration and intensity of metabolic exposure, details of renal phenotype, and preanalytical sample handling procedures may also affect MaR1 levels. The storage conditions and the absence of a formal stability assessment for MaR1 should be considered when interpreting the measured serum concentrations. Therefore, despite controlled handling and limitation to a single freeze–thaw cycle, storage-related degradation cannot be excluded and may have influenced the measured MaR1 concentrations. Proteinuria was assessed using a single UPCR measurement rather than repeated measurements or a complete DKD staging framework. These limitations do not invalidate the healthy control–T2DM separation. However, they limit the interpretation of MaR1 as a complication-specific biomarker.

These findings indicate that serum MaR1 should be interpreted within a systemic MaR1 difference that may occur in T2DM independently of microvascular complication stage. This study extends the human MaR1 literature across DR phenotypes and proteinuria. It also shows that lower serum MaR1 should not be interpreted as a change specific to advanced retinopathy or renal protein loss. The clinical meaning of serum MaR1 in retinal and renal microvascular disease should be clarified in larger cohorts, prospective studies, and designs that evaluate serum or plasma, ocular fluid, and urinary samples together.

## 5. Conclusions

This study positions serum MaR1 as the human serum correlate of a systemic MaR1 difference associated with diabetes, rather than as a measure that directly tracks retinopathy stage or proteinuria level in T2DM. This interpretation indicates that the clinical meaning of MaR1 in T2DM cannot be read solely through retinal staging or proteinuria. Instead, MaR1 should be evaluated within a broader biomarker framework that considers systemic, ocular, and renal compartments together. The true clinical value of serum MaR1 in T2DM should be clarified in larger prospective studies that jointly assess complication phenotypes and biological matrices.

## Figures and Tables

**Table 1:** Baseline clinical and biochemical characteristics across the four study groups.

Characteristic	Healthy Control	T2DM Without DR	T2DM with NPDR	T2DM with PDR	*p*
Age, years	45.7 (15.2)	58.2 (9.71)	63.2 (11.7)	62.4 (8.07)	<0.001
Female sex, *n* (%)	7 (35.0)	17 (53.1)	11 (52.4)	9 (45.0)	0.591
Body mass index, kg/m^2^	27.0 (24.6–28.4)	26.5 (24.5–32.8)	26.8 (25.9–31.1)	29.0 (26.3–33.8)	0.369
Diabetes duration, years	Not applicable	7.00 (3.75–11.2)	13.0 (7.00–20.0)	15.0 (10.0–21.2)	<0.01
Fasting glucose, mg/dL	87.0 (80.8–89.0)	158 (125–201)	219 (166–286)	182 (140–230)	<0.001
HbA1c, %	5.45 (5.30–5.62)	7.40 (7.05–9.65)	8.40 (7.40–10.2)	9.35 (8.53–10.2)	<0.001
C-peptide, ng/mL	2.21 (1.77–2.90)	1.94 (1.56–2.53)	1.92 (1.26–3.94)	2.25 (1.26–3.20)	0.838
Triglycerides, mg/dL	115 (90.8–162)	204 (142–256)	128 (107–254)	198 (130–236)	<0.01
LDL-C, mg/dL	121 (40.9)	122 (31.5)	107 (38.1)	116 (42.5)	0.491
HDL-C, mg/dL	47.5 (42.0–54.5)	46.5 (40.8–55.5)	46.0 (42.0–56.0)	52.0 (40.5–58.8)	0.920
Total cholesterol, mg/dL	195 (46.4)	213 (47.3)	190 (47.0)	212 (44.6)	0.236
Urea, mg/dL	25.0 (21.5–30.0)	30.0 (25.5–36.2)	36.0 (30.0–40.0)	29.5 (27.8–44.2)	<0.01
Creatinine, mg/dL	0.655 (0.568–0.765)	0.755 (0.627–0.860)	0.790 (0.660–0.890)	0.830 (0.623–0.942)	0.066
Urine protein-to-creatinine ratio	0.091 (0.087–0.098)	0.157 (0.087–0.220)	0.186 (0.101–0.398)	0.243 (0.182–0.670)	<0.01
Serum Maresin-1	89.8 (82.8–97.2)	34.3 (33.0–36.0)	35.8 (34.4–36.7)	34.0 (33.1–35.7)	<0.001

Values are mean (SD), median (IQR), or *n* (%). Welch ANOVA was used for age. One-way ANOVA was used for LDL-C and total cholesterol. Kruskal–Wallis tests were used for BMI, diabetes duration, fasting glucose, HbA1c, C-peptide, triglycerides, HDL-C, urea, creatinine, urine protein-to-creatinine ratio, and serum Maresin-1. Pearson chi-square test was used for female sex. Diabetes duration was compared only across the three T2DM groups.

**Table 2:** Serum MaR1 levels across the four study groups.

Group	*n*	Serum Maresin-1, Median (IQR)	Range
Healthy control	20	89.8 (82.8–97.2)	66.0–310
T2DM without DR	32	34.3 (33.0–36.0)	31.4–45.0
T2DM with NPDR	21	35.8 (34.4–36.7)	33.5–41.9
T2DM with PDR	20	34.0 (33.1–35.7)	32.0–49.0

Kruskal–Wallis test: H = 49.36, *p* < 0.001, epsilon-squared = 0.521.

**Table 3:** Holm-adjusted pairwise comparisons for serum MaR1.

Comparison	Group 1 Median (IQR)	Group 2 Median (IQR)	Median Difference	Holm-Adjusted *p*
Healthy control vs. T2DM without DR	89.8 (82.8–97.2)	34.3 (33.0–36.0)	55.55	<0.001
Healthy control vs. T2DM with NPDR	89.8 (82.8–97.2)	35.8 (34.4–36.7)	54.05	<0.001
Healthy control vs. T2DM with PDR	89.8 (82.8–97.2)	34.0 (33.1–35.7)	55.90	<0.001
T2DM without DR vs. T2DM with NPDR	34.3 (33.0–36.0)	35.8 (34.4–36.7)	−1.50	0.381
T2DM without DR vs. T2DM with PDR	34.3 (33.0–36.0)	34.0 (33.1–35.7)	0.35	0.745
T2DM with NPDR vs. T2DMDR vs. T2DM with PDR	35.8 (34.4–36.7)	34.0 (33.1–35.7)	1.85	0.381

Post hoc comparisons were performed using Dunn-type rank-based pairwise comparisons with Holm adjustment. Median difference is Group 1 minus Group 2.

**Table 4:** Prespecified MaR1–proteinuria correlations.

Analysis Set	*n*	Variable	Spearman Rho	*p*
All participants	93	Urine protein-to-creatinine ratio	−0.272	0.008
T2DM only	73	Urine protein-to-creatinine ratio	−0.057	0.634

Spearman rank correlation was used. The all-participant estimate should be interpreted alongside group separation because healthy controls and T2DM phenotypes occupy different clinical strata.

**Table 5:** T2DM-only correlations of serum MaR1 with selected clinical and biochemical variables.

Variable	*n*	Spearman Rho	*p*	FDR q
Urine protein-to-creatinine ratio	73	−0.057	0.634	0.863
Urea	73	0.080	0.501	0.863
Creatinine	73	0.197	0.095	0.465
Age	73	−0.185	0.116	0.465
Body mass index	73	0.081	0.494	0.863
Diabetes duration	73	−0.037	0.753	0.863
Fasting glucose	73	−0.011	0.926	0.926
HbA1c	73	−0.037	0.755	0.863

Spearman rank correlation was used within the T2DM-only analysis set. FDR q values were calculated using the Benjamini–Hochberg method across the variables shown in this table.

**Table 6:** Supportive regression models for the association between proteinuria and serum MaR1.

Model	*n*	β per 1 SD Proteinuria (95% CI)	*p*	Adjusted R^2^
All participants: unadjusted	93	−3.84 (−7.75 to 0.08)	0.055	0.001
All participants: adjusted for age, sex, and BMI	93	−2.80 (−7.31 to 1.71)	0.221	0.159
All participants: adjusted for age, sex, BMI, and study group	93	0.25 (−2.49 to 2.99)	0.856	0.547
T2DM only: unadjusted	73	0.64 (−1.41 to 2.69)	0.537	0.024
T2DM only: adjusted for age, sex, BMI, HbA1c, and diabetes duration	73	0.66 (−1.31 to 2.62)	0.508	0.073
T2DM only: adjusted for age, sex, BMI, HbA1c, diabetes duration, and DR phenotype	73	0.82 (−1.26 to 2.90)	0.436	0.071

Outcome: raw serum Maresin-1. Main exposure: urine protein-to-creatinine ratio standardized per 1 SD. CIs and *p* values are based on HC3 robust standard errors because diagnostic checks identified influential observations and heteroscedasticity in the raw OLS models. These models are supportive and should not replace the primary non-parametric analyses.

**Table 7:** Supportive age-, sex-, and BMI-adjusted group model for serum Maresin-1.

Term	β (95% CI), pg/mL	*p*
T2DM without DR vs. healthy control	−62.64 (−85.40 to −39.87)	<0.001
T2DM with NPDR vs. healthy control	−61.07 (−82.39 to −39.75)	<0.001
T2DM with PDR vs. healthy control	−61.73 (−83.16 to −40.30)	<0.001
Age, per year	−0.27 (−0.64 to 0.09)	0.146
Male vs female	−6.59 (−21.51 to 8.33)	0.387
BMI, per kg/m^2^	0.25 (−0.40 to 0.91)	0.450

Outcome: raw serum MaR1. The model was specified as MaR1 ~ study group + age + sex + BMI. The reference categories were healthy control for study group and female for sex. Inference was based on HC3 robust standard errors. This model was supportive and should not replace the primary Kruskal–Wallis analysis.

## Data Availability

The original contributions presented in this study have been included in the article. For further information, please contact the corresponding author.

## Abbreviations

The following abbreviations are used in this manuscript:

DR	Diabetic retinopathy
ELISA	Enzyme-linked immunosorbent assay
HbA1c	Glycated hemoglobin
HDL-C	High-density lipoprotein cholesterol
IQR	Interquartile range
LDL-C	Low-density lipoprotein cholesterol
MaR1	Maresin-1
NPDR	Non-proliferative diabetic retinopathy
PDR	Proliferative diabetic retinopathy
SD	Standard deviation
T2DM	Type 2 diabetes mellitus

## Appendix A

### Supportive T2DM-Only Phenotype Analysis

Among participants with T2DM, serum Maresin-1 did not differ significantly across no DR, non-proliferative DR, and proliferative DR phenotypes (Kruskal–Wallis H = 4.44, *p* = 0.109, epsilon-squared = 0.035). The ordinal association between DR stage code and serum Maresin-1 was not significant (Spearman rho = −0.001, *p* = 0.996).

**Table A1:** Serum Maresin-1 across T2DM retinopathy phenotypes.

T2DM Phenotype	*n*	Serum Maresin-1, Median (IQR)	Range
No DR	32	34.3 (33.0–36.0)	31.4–45.0
Non-proliferative DR	21	35.8 (34.4–36.7)	33.5–41.9
Proliferative DR	20	34.0 (33.1–35.7)	32.0–49.0

Kruskal–Wallis test across T2DM phenotypes: H = 4.44, *p* = 0.109, epsilon-squared = 0.035.

**Table A2:** Holm-adjusted pairwise comparisons across T2DM phenotypes.

Comparison	Group 1 Median (IQR)	Group 2 Median (IQR)	Median Difference	Holm-Adjusted *p*
No DR vs. Non-proliferative DR	34.3 (33.0–36.0)	35.8 (34.4–36.7)	−1.50	0.163
No DR vs. Proliferative DR	34.3 (33.0–36.0)	34.0 (33.1–35.7)	0.35	0.679
Non-proliferative DR vs. Proliferative DR	35.8 (34.4–36.7)	34.0 (33.1–35.7)	1.85	0.156

Post hoc comparisons were performed only as supportive analyses. Median difference is Group 1 minus Group 2.
